# Editorial Comment to A case in which bladder cancer invaded the ureteral orifice and was resected via photodynamic diagnosis‐assisted transurethral resection involving orally administered 5‐aminolevulinic acid

**DOI:** 10.1002/iju5.12114

**Published:** 2019-09-16

**Authors:** Yasushi Nakai, Kiyohide Fujimoto

**Affiliations:** ^1^ Department of Urology Nara Medical University Kashihara Japan

Watanabe *et al*.^1^ reported an interesting case of bladder cancer in the intramural ureter exhibiting red fluorescence with the use of orally administered 5‐aminolevulinic acid‐mediated photodynamic diagnosis (ALA‐PDD). The tumor was resected with negative margins, which did not show red fluorescence by ALA‐PDD. The present study reports that an exact diagnosis and precise resection of the bladder tumor in the intramural ureter are possible with the use of ALA‐PDD. Faba *et al*.^2^ reported a good oncological outcome for conventional transurethral resection of bladder cancer without T1 or carcinoma *in situ* in the intramural ureter. ALA‐PDD can be considered in patients suspicious for bladder cancer in the intramural ureter, though long‐term follow‐up for local or upper urinary tract recurrence is needed in the present case, and accumulating information on such cases is necessary.

False‐positive results should be noted during ALA‐PDD for bladder cancer.^3^ Oblique illumination and inflammation can lead to false‐positive results, which are often observed at the bladder neck, trigone, and around the orifice.^4^ Because ureteral stenosis can occur after resection of the orifice (11.6%),^2^ unnecessary surgery should be avoided. Draga *et al*.^5^ reported that the learning curve for surgeons performing transurethral resection under PDD was proportional to the decrease in the number of false positives up to 12–18 months after the initial PDD procedure. Thus, some experience with ALA‐PDD and careful observation are necessary to perform ALA‐PDD for bladder tumors in the intramural ureter.

The present case also reveals the potential of ALA‐PDD for diagnosis of upper urinary urothelial carcinoma (UTUC). Kata *et al*.^6^ reported the potential of ureteroscopy using 5‐ALA, and that ALA‐PDD for UTUC can identify tiny lesions and carcinoma *in situ*, which is missed under conventional white light. 5‐ALA can be administered using either an oral or transurethral route. On the other hand, hexaminolevulinate, which is used in Europe for PDD of non–muscle‐invasive bladder cancer, is administered via intravesical instillation only. Therefore, ALA‐PDD is a promising diagnostic tool for various kinds of cancers including UTUC, and trials of ALA‐PDD in the treatment of such cancers are being performed.

The use of ALA‐PDD for bladder cancer has recently been approved in Japan and performed in various institutions. We should report the effectiveness of ALA‐PDD from Japan, and transurethral resection with ALA‐PDD should be approved as a treatment for bladder carcinoma outside of Japan as emphasized by Watanabe *et al*. in their keynote message.

## Conflict of interest

The authors declare no conflict of interest.
